# Antioxidant and anti-hepatitis A virus activities of *Ecklonia cava* Kjellman extracts

**DOI:** 10.1016/j.heliyon.2024.e25600

**Published:** 2024-02-01

**Authors:** Ye-Sol Kim, Ki An Kim, Hye-Young Seo, Sung Hyun Kim, Hee Min Lee

**Affiliations:** a Kimchi Industry Promotion Division, World Institute of Kimchi, Gwangju, 61755, South Korea; b Marine Biotechnology Research Center, Jeonnam Bioindustry Foundation, Jeollanam-do, 59108, South Korea; c Pulmuone Institute of Technology, Cheongju, 28164, South Korea; d Department of Food Science & Technology, Chonnam National University, Gwangju, 61186, South Korea

**Keywords:** Hepatitis A virus, *Ecklonia cava*, Antioxidant activity, Antiviral, Untargeted metabolomics

## Abstract

*Ecklonia cava* is a nutrient-rich algae species that contains abundant physiological phytochemicals, including peptides, carotenoids, fucoidans, and phlorotannins. However, elucidation of the antiviral effects of this algae and identification of new functional ingredients warrant further investigation. This study was aimed at investigating the potential anti-hepatitis A virus activities of extracts of *E. cava* prepared in different solvents. *E. cava* extracts were prepared using hot water and 70 % ethanol. The antioxidant activities of the extracts were confirmed by analyzing the total phenolic content, as well as 2,2-diphenyl-1-picrylhydrazyl and 2,2′-azino-bis-3-ethylbenzothiazoline-6-sulfonic acid radical scavenging activities. The inhibitory effects of the extracts against hepatitis A virus were analyzed using real-time polymerase chain reaction. The *E. cava* extract yield was 22.5–27.2 % depending on the extraction solvent. The 2,2-diphenyl-1-picrylhydrazyl radical scavenging activity was 70.44 % and 91.05 % for hot water and ethanol extracts at a concentration of 1000 ppm. The 2,2′-azino-bis-3-ethylbenzothiazoline-6-sulfonic acid radical scavenging activity of the ethanol extract was the highest (93.57 %) at 1000 μg/mL. Fourier-transform infrared was used to identify the functional groups (phlorotannin and alginate) in the extraction solvents. Ultra-high performance liquid chromatography with quadrupole time-of-flight tandem mass spectrometry analysis revealed a potential bioactive compound previously unidentified in *E. cava*. Finally, we identified the antiviral activity of *E. cava* extracts against hepatitis A virus replication. These findings demonstrate that *E. cava* could be used as an anti-hepatitis A virus functional food and biological material.

## Highlights

• *Ecklonia cava* contains abundant phytochemicals with health benefits.
• This algal species demonstrated antiviral properties against hepatitis A virus.
• We found a new potential bioactive compound in *E. cava.*
• *E. cava* has potential medicinal applications.

## 1 Introduction

*Ecklonia cava* Kjellman (*E. cava*) is a brown alga that belongs to the class Phaeophyceae and family Laminariaceae. This perennial plant grows in rocky coastal areas, especially in Korea and Japan [1]. *E. cava* has garnered interest as a food and as a medicinal plant for treatment of inflammation, diabetes, fever, and cancer [[2], [3], [4], [5]]. The cell wall of filamentous *E. cava* macroalgae contains sulfated polysaccharides [6]. Moreover, this alga is nutrient-dense, containing abundant phytochemicals, such as phlorotannins, sulfated polysaccharides (i.e., fucoidans), peptides, and carotenoids. Phlorotannins that are composed of phloroglucinol monomers, a type of benzenetriol, vary in their degree of polymerization and bonding. Furthermore, phlorotannins identified from *E. cava* reportedly include dieckol, phlorofucofuroeckol, 6,6′-bieckol, eckol, eckstolonol, phlorofucofuroeckol A, 7-phloroeckol, 6,6′-bieckol, 8,8′-bieckol, and triphlorethol A [[7], [8], [9]]. These compounds possess antioxidant, anti-inflammatory, anti-allergic, anti-diabetic, anti-hypnotic, and neuroprotective properties. Sulfated polysaccharides exhibit antioxidant, anti-cancer, anti-coagulant, anti-hyperlipidemic, and antiviral activities [10]. In addition, phlorotannins from marine algae have been studied for their anti-proliferative, antihypertensive, anti-inflammatory, and anti-diabetic properties [[11], [12], [13], [14], [15]]. However, only a few studies have described the antiviral effects of *E. cava* and the biological mechanisms underlying such effects [[16], [17], [18]].

Phlorotannin is extracted using water, which is the safest solvent according to the green chemical principle. However, its yield is low compared with that obtained when using organic solvents. It is not clear whether organic solvents promote the extraction of unbound phlorotannins [19] or reduce the formation of phlorotannin complex after extraction [20,21].

Hepatitis A virus (HAV) is a common causative agent of acute viral hepatitis worldwide and can be caused by foodborne and waterborne transmission. HAV infects the liver follows the food absorption route [22]. The prevalence of hepatitis A virus (HAV) infection has decreased in developed countries due to the development of vaccines and widespread vaccination. Nonetheless, many people are infected with this virus in developing and underdeveloped countries where adequate sanitation and clean water facilities are lacking [23]. Older adults have a higher incidence of infection compared with young children [24]. Although prevention through timely vaccination and diagnosis is the top priority, it is also important to investigate the infection mechanism of this virus and devise treatment strategies for rapid recovery. Interestingly, the plants studies have considerably increased [25,26] and the use of natural products as anti-HAV treatments is increasing, and several studies have been published on these. Zinc compounds [22], essential oils [27,28], grape seed [29,30], *Thalassodendron ciliatum* [31], and Japanese rice-koji miso [32] have been shown to exhibit antiviral activity against HAV replication. However, there have been no reports on the effects of seaweed extract against this virus. Therefore, this study was conducted to demonstrate that crude polysaccharides and phenolic compounds in water and 70 % ethanol extracts of *E. cava* contain abundant fucose and sulfated groups and exert biological activity against HAV.

## 2 Methods

### 2.1 *E. cava* extract

*E. cava* was frozen in a −70 °C deep freezer and freeze-dried using a freeze dryer (FD8508, Ilshin Biobase Co., Ltd, Dongducheon, Korea). The dried and pulverized samples were extracted with hot water or 70 % ethanol, concentrated using a rotary evaporator, and stored at 4 °C for experimentation. The extraction yield is expressed as a percentage of the extract weight relative to the sample dry weight.

Dried *E. cava* powder (300 g) was extracted using distilled water or 70 % ethanol. Hot water extraction (1.5 L; 1:15, w/v) was carried out twice at 100 °C for 3 h. Similarly, 70 % ethanol extraction (1.0 L; 1:10, w/v) was carried out twice at 70 °C for 3 h using a reflux cooling extractor. The extract was filtered through Whatman No. 4 filter paper, and the filtrate was concentrated via vacuum evaporation at 40 °C. The extract was freeze-vacuum-dried and stored at −80 °C until further use.

(1) $$\text{Extraction}\;\text{yield}=\frac{Weight\;of\;solids\;before\;extraction}{Weight\;of\;sample\;before\;extraction}$$

### 2.2 Quantification of total phenolic content

Total phenolic content was measured using the Folin–Denis method [33]. Here, 0.2 mL E*. cava* extract and 0.2 mL Folin reagent were mixed and reacted at room temperature for 3 min. Then, 0.4 mL of a 10 % sodium carbonate solution was mixed and allowed to react in the dark for 40 min. Absorbance of the mixture was measured at 760 nm using a microplate reader (SpectraMax i3, Molecular Devices, Sunnyvale, CA, USA). Phenolic content was quantified based on the calibration curve prepared using gallic acid standard at 125, 250, 500, and 1000 μg/mL. The results are expressed as gallic acid equivalents (GAE) in mg/g of dry *E. cava* weight. The experiment was replicated three times, and data are presented as mean ± standard deviation (SD).

### 2.3 Total flavonoid content

The flavonoid content of *E. cava* was measured as described previously [34], with some modifications. Briefly, 0.5 mL E*. cava* extract was mixed with 0.5 mL diethylene glycol. Then, 10 μL of 1 N NaOH was added to the mixture, thoroughly mixed, and reacted at 37 °C for 1 h. The absorbance was measured at 420 nm using a microplate reader. Total flavonoid content was quantified based on the calibration curve prepared using quercetin standard at 125, 250, 500, and 1000 μg/mL. The results are expressed as quercetin equivalents (QE) in mg/g of dry *E. cava* weight. The experiment was conducted in triplicates, and data are presented as mean ± SD.

### 2.4 2,2-Diphenyl-1-picrylhydrazyl (DPPH) radical scavenging activity

The DPPH radical scavenging activity of *E. cava* was assessed using the Blois method [35]. Briefly, 0.1 mL of each sample at 50, 125, 250, 500, and 1000 μg/mL was thoroughly mixed with 0.9 mL of freshly prepared ethanolic solution of DPPH (0.2 mM). The reaction mixture was incubated at 37 °C for 30 min, and absorbance was measured at 590 nm using a microplate reader. The sample concentration corresponding to 50 % DPPH radical scavenging activity was calculated and expressed as an IC_50_ value (μg/mL). The experiment was conducted in triplicates, and data are reported as mean ± SD. The DPPH radical scavenging activity was calculated as a percentage using the following formula:

(2) $$\text{DPPH}\;\text{radical}\;\text{scavenging}\;\text{activity}\;\left(\%\right)=1\;-\;\left({\text{Abs}}_{\text{sample}}\;/\;{\text{Abs}}_{\text{blank}}\right)\times 100$$

### 2.5 2,2′-azino-bis-3-ethylbenzothiazoline-6-sulphonic acid (ABTS) radical scavenging activity

ABTS radical scavenging activity was evaluated as described previously [36], incorporating slight modifications. Equal amounts of ABTS (7.4 mM) and potassium persulfate (2.6 mM) solutions were mixed and allowed to react in the dark for 24 h to generate ABTS free radicals. After 24 h, the ABTS radical solution was diluted with methanol to obtain an absorbance of 0.7–1.0 ± 0.02 at 734 nm. Then, 0.1 mL each of 10, 25, 50, 125, and 250 μg/mL samples was thoroughly mixed with 0.9 mL of freshly prepared ABTS radical solution in methanol. The mixture was reacted at 37 °C for 30 min, and absorbance was measured at 734 nm using a microplate reader. The sample concentration corresponding to 50 % ABTS radical scavenging activity was calculated and expressed as an IC_50_ value (μg/mL). The experiment was repeated three times, and the results are expressed as mean ± SD. The ABTS radical scavenging activity was determined as a percentage using the formula:

(3) $$\text{ABTS}\;\text{radical}\;\text{scavenging}\;\text{activity}\;\left(\%\right)=\left(1\;-\;{\text{Abs}}_{\text{sample}}\;/\;{\text{Abs}}_{\text{blank}}\right)\times 100$$

### 2.6 Fourier-transform infrared (FTIR) spectra

The IR spectrum of *E. cava* was recorded in the 4000–400 cm^−1^ range using a Thermo Scientific (Waltham, MA, USA) Nicolet iS50 FTIR spectrometer. FT-Raman spectra were recorded (512 scans/spectrum) on a Thermo Scientific Nicolet iS50 FTIR spectrometer equipped with an iS50 Raman Module using an Nd:YAG laser with an excitation wavelength of 1064 nm operating at 500 mW and an InGaAs detector.

The FTIR spectral analysis of *E. cava* samples was performed over a spectral range of 400–4000 cm^−1^, with a resolution of 4 cm^−1^. Each spectrum was constructed from 16 scans in the absorbance mode, recording over the 3500–500 cm^−1^ region.

### 2.7 Ultra-high performance liquid chromatography with quadrupole time-of-flight tandem mass spectrometry (UPLC-QTOF-MS)

UPLC was performed on a Waters (Milford, MA, USA) ACQUITY UPLC system using an ACQUITY UPLC HSS T3 column (100 mm × 2.1 mm, 1.8 μm; Waters) maintained at an oven temperature of 40 °C. The mobile phase, comprising solvent A (0.1 % formic acid in water) and solvent B (0.1 % formic acid with acetonitrile), was delivered at a flow rate of 0.5 mL/min. The elution gradient was set as follows: 0–5 min, 3 % phase B; 5–16 min, 3–100 % phase B; 16–17 min, 100 % phase B; 17–19 min, 100–3 % phase B; and 19–20 min, 3 % phase B. MS detection was carried out using a SYNAPT G2-Si HDMS QTOF mass spectrometer (Waters) with electrospray ionization. The MS detector conditions were as follows: ESI-positive capillary voltage, 3 kV; negative capillary voltage, 2 kV; cone voltage, 40 V; source temperature, 120 °C; and desolvation temperature, 500 °C. The MS/MS data were obtained using a collision energy ramp from 20 to 40 eV in the MS^E^ mode. The scanning time was 0.2 s, with a mass range *m*/*z* of 50–1200 Da. A solution of leucine encephalin, sprayed at a flow rate of 10 μL/min, served as a reference ion for both positive (*m*/*z* 556.2771) and negative (*m*/*z* 554.2615) ion modes. Data acquisition and analysis were managed using the UNIFI V1.71 software (Waters). Identification of peaks was carried out by screening against the propriety scientific library of UNIFI V1.71.

### 2.8 Cell culture

HepG2 cells were obtained from the Korean Cell Line Bank (Seoul, Korea) and cultured in Dulbecco's modified Eagle medium (Welgene, Daegu, Korea). The medium was supplemented with 10 % (v/v) fetal bovine serum, 100 IU/mL penicillin, and 100 μg/mL streptomycin and the culture was performed under 5 % CO_2_ at 37 °C.

### 2.9 CCK-8 assay

HepG2 cells were enumerated and seeded at approximately 4000 cells/well in a 96-well cell culture plate (Corning Inc., Corning, NY, USA). After 48 h of incubation at 37 °C in a 5 % CO_2_ humidified atmosphere, the culture medium was substituted with a series of concentrations of extracts diluted in the corresponding culture fluid. Thereafter, 10 μL of the CCK-8 reagent (Dogindo, Japan) was added to each well. Finally, the optical density at 450 nm was quantified using a microplate reader after 2 h incubation at 37 °C. Cell viability is presented as the percentage of each concentration accounted for by the control.

### 2.10 Infection of cells with HAV

HepG2 cells were seeded 20 h prior to infection in 12-well plates at a density of 2.5 × 10^5^ cells/well. One hour prior to infection, complete medium was replaced with the medium containing 1 % FBS. The cells were inoculated with HAV diluted in the medium containing 1 % FBS at a multiplicity of infection of 1 (for HAV, HM175 strain). Three hours after infection, the cells were washed with phosphate-buffered saline, the medium was exchanged with complete medium, and *E. cava* extract was added.

### 2.11 Real-time polymerase chain reaction (PCR)

Total ribonucleic acid (RNA) was isolated using the RNeasy Plus Mini Kit (Qiagen, Valencia, USA) and transcribed into complementary deoxyribonucleic acid (cDNA) using a reverse transcriptase kit (Thermo Fisher Scientific). Real-time PCR was performed using the SYBR green method (SsoAdvanced Universal SYBR Green Super mix; Bio-Rad, Hercules, CA, USA) on an ABI-7500 FAST Real-Time PCR System (Applied Biosystems, Foster City, CA, USA). Primers were specific for the housekeeping genes β-actin and *HAV* (Table 1). The first step in the PCR amplification was at 98 °C for 3 min, followed by 40 repetitive cycles using SYBR Green (98 °C for 15 s, 60 °C for 30 s). At the end of the program, melting curve analysis was performed over a temperature range from 60 to 95 °C, and fluorescence data were acquired at every 0.3 °C increase in temperature.

**Table 1:** Primers used to detect gene expression at the mRNA level.

Gene name	Primer sequences (5′–3′)
***β-actin***	**F: CCA CCA TGT ACC CTG GCA TT** **R: CGG ACT CGT CAT ACT CCT GC**
***HAV***	**F: GCG GCG GAT ATT GGT GAG** **R: CAA TGC ATC CAC TGG ATG AGT**

### 2.12 Statistical analyses

All experimental results are expressed as means ± standard deviation of values from three repeated measurements. Statistical analyses were performed using the SPSS software package (Statistical Package for the Social Science, version 19, SPSS Inc., Chicago, IL, USA). Student's *t*-test and two-way analysis of variance (ANOVA) were performed to evaluate the antioxidant and anti-HAV properties of the *E. cava* extracts. Statistical significance was set at *p* < 0.05.

## 3 Results and discussion

### 3.1 Crude extract yield and bioactivity characterization

Crude phenolic and flavonoid compounds were extracted from 300 g of *E. cava* powder using hot water or 70 % ethanol, and their dry weights were 81.7 and 67.5 g, respectively. As shown in Table 2, the antioxidant capacity of the 70 % ethanol extract was higher than that of the hot water extract with regard to the total phenolic and flavonoid content. The phenolic content in the hot water and ethanol extracts were 124 and 205 mg GAE/g, respectively, and the flavonoid content was 77.0 and 118.0 mg QE/g, respectively. Moreover, the hot water extract showed a higher extraction yield than the ethanol extract. Ethanol extract had higher total phenolic and flavonoid content than hot water extract despite its lower extraction yield. Similarly, Lee et al. [37] reported a higher water extraction yield from *E. cava* compared with that achieved using 100 % methanol, whereas the total phenolic content was much higher in the methanol extract. In addition, Liu et al. [38] reported that lower ethanol concentrations and elevated temperatures were associated with an increase in the extraction yield.

**Table 2:** Comparison of extraction yield, as well as total phenolic and flavonoid content of *Ecklonia cava* extracts.

	Hot water	70 % ethanol
Extraction yield (%)	27.2 ± 1.7^a^	22.5 ± 1.2
Total phenolic content (mg GAE^b^/g)	124 ± 1.0	205 ± 0.2
Total flavonoid content (mg QE^c^/g)	77.0 ± 1.0	118 ± 1.5

a Data are expressed as means ± SD (*n* = 3).

b GAE, gallic acid equivalent.

c QE, quercetin equivalent.

### 3.2 In vitro antioxidant activity of phenolic compounds

**Fig. 1:**
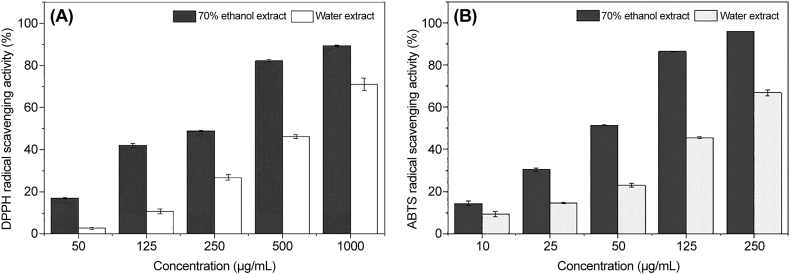
Antioxidant activity of *Ecklonia cava* water and 70 % ethanol extracts. (A) DPPH radical scavenging activity. (B) ABTS radical scavenging activity. Data are expressed as mean ± SD (*n* = 3).

The antioxidant activities of the two extracts were evaluated and compared using DPPH and ABTS scavenging assays (Fig. 1). The %scavenging activity of the two extracts in these assays exhibited an exponential increase in a dose-dependent manner. The IC_50_ values for ethanol extract activity against DPPH and ABTS were 238.9 and 55.4 μg/mL, respectively. The ethanol extract was more proficient in scavenging these radicals than the hot water extract. Liu et al. [38] reported that higher ethanol concentrations corresponded to stronger antioxidant activity. Using the response surface methodology, they determined the optimal conditions to be 80 % ethanol, 20 °C extraction temperature, and a solvent-to-solid ratio of 70 mL/g.

### 3.3 FTIR spectra

**Fig. 2:**
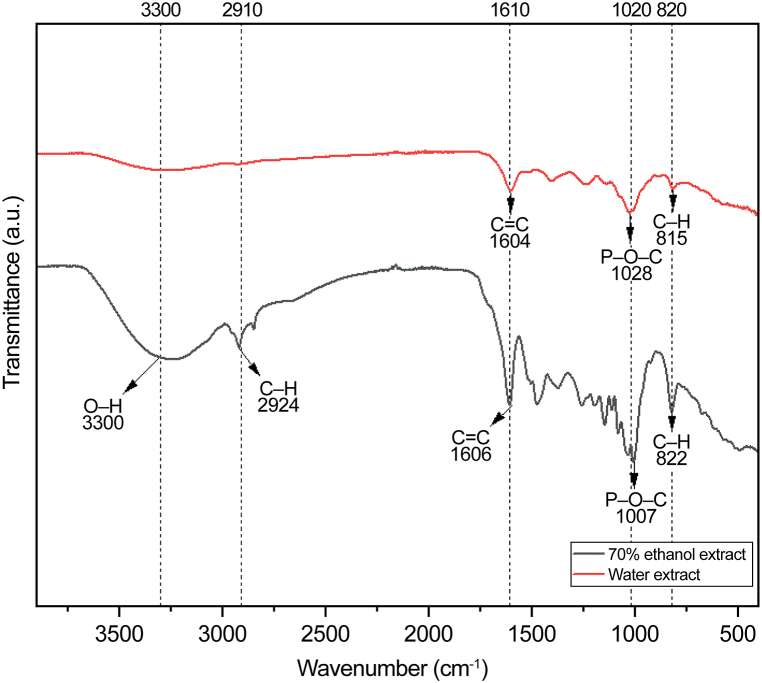
FTIR spectra of 70 % ethanol and hot water extracts from *Ecklonia cava*.

FTIR spectroscopy was used to evaluate significant differences between the analyzed extracts, including the number of peaks due to chemical reactions, peak intensities, and peak shapes due to differences in the composition. *E. cava* extracts were analyzed in the 4000–500 cm^−1^ range using FTIR spectroscopy to determine the specific absorption bands (Fig. 2). There were more than five peaks for each extract, indicating that the analyzed chemicals were complex. The peaks at approximately 3300 and 2910 cm^−1^ were attributed to the vibrational stretches of the intermolecular and intramolecular hydroxyl groups, respectively. Therefore, these peaks are often attributed to the strength of hydrogen bonds in polar molecules [39]. The peaks at approximately 3300 and 2910 cm^−1^ in the ethanol extract were labeled as H–*O*–H bending vibrations due to O–H and C–H stretching. Furthermore, they were attributed to hydrogen bonding between phlorotannin and water, forming adducts. The peak at approximately 1610 cm^−1^ characterizes C=C bond stretching vibrations in aromatic rings with signals from carboxylic acids, aldehydes, and ketones [40]. This was indicative of phlorotannins and alginate oligomers within the extract [41]. The 1610 cm^−1^ peak exhibited reduced intensity in the hot water extract compared with that in the ethanol extract. Additionally, the peak at approximately 820 cm^−1^ was attributed to C–H stretching vibrations, displaying higher intensity in the ethanol extract than in the hot water extract. These results imply a significant presence of dieckol and other phenolic compounds in the ethanol extracts.

### 3.4 Tentative identification of compounds using UPLC-MS/MS

Qualitative analysis and identification of the phenolic compounds from *E. cava* were carried out using UPLC-ESI-QTOF-MS/MS in both the positive and negative ionization modes. The criteria for further compound analysis were a mass error of -3–3 ppm and response value > 1000. A total of 25 compounds were identified in the *E. cava* extracts (Table 3), including phlorotannins, phenolic acids, terpenes, and organic acids. Predominantly, these peaks were tentatively assigned to phlorotannin derivatives, such as dieckol, eckol, 6,6′-bieckol, fucoxanthin, and phlorofucofuroeckol A. The compounds with relatively higher content in the ethanol extract comprised 6,6′-bieckol, dieckol, genkwadaphnin, and pyropheophorbide A. Phlorotannins contained in seaweeds, especially brown algae such as *E. cava* [3], *Ecklonia arborea* [42], *Fucus spiralis*, *Macrocystis pyrifera*, *Laminaria digitata*, and *Sargassum fusiforme* have antioxidant properties [43]. Furthermore, the compounds exhibiting higher content in the ethanol extract compared with that in the hot water extract included 6,6′-bieckol, dieckol, genkwadaphnin, and pyropheophorbide A. Wu et al. [44] reported the greatest accumulation of free phenolic compounds in 70 % ethanol extract of *Sargassum polycystum* compared with that in extracts prepared in various extraction solvents. Furthermore, the compounds identified in this study, such as chicoric acid [45], abietic acid [46], pyropheophorbide A [47], and taurocholic acid [48], are recognized for their promising antioxidant and antiviral activities.

**Table 3:** Compounds identified in *Ecklonia cava* extracts using UPLC/Q-TOF MS/MS.

No.	Electrospray	Component name	Formula		RT	Observed *m*/*z*	Error	Adducts
1	ES+	Chicoric acid	C_22_H_18_O_12_		3.26	497.0705	3	+Na
2	ES+	Dihydroactinidiolide	C_11_H_16_O_2_		6.17	181.1218	−2.8	+H
3	ES+	1-Monopalmitin	C_19_H_38_O_4_		8.94	331.2847	1.1	+H
4	ES+	Retinol	C_20_H_30_O		9.21	287.2374	1.7	+H
5	ES+	(−)-Abietic acid	C_20_H_30_O_2_		9.24	303.2322	1.2	+H
6	ES+	Palmitoleic acid	C_16_H_30_O_2_		9.34	277.2145	2.6	+Na
7	ES+	Butyl isobutyl phthalate	C_16_H_22_O_4_		9.57	301.1402	−2.7	+Na
8	ES+	Linolenic acid	C_18_H_30_O_2_		9.86	301.2131	−2.3	+Na
9	ES+	Genkwadaphnin	C_34_H_34_O_10_		11.21	603.2228	0.6	+H, +Na
10	ES+	Fucoxanthin	C_42_H_58_O_6_		11.31	681.4127	0.2	+Na, +H
11	ES+	Pyrophaeophorbide A	C_33_H_34_N_4_O_3_		11.98	535.2688	−2.9	+H, +Na
12	ES+	Taraxerone	C_30_H_48_O		12.45	425.3768	−2.4	+H
13	ES+	Bis(2-ethylhexyl)phthalate	C_24_H_38_O_4_		12.69	413.2659	−0.8	+Na, +H
14	ES+	Stigmast-4-ene-3,6-dione	C_29_H_46_O_2_		12.76	427.3562	−2.1	+H
15	ES+	Daturametelin H	C_34_H_46_O_9_		12.86	621.305	2.6	+Na
16	ES-	Dieckol	C_36_H_22_O_18_		0.58	741.0727	−0.9	- H, +HCOO
17	ES-	Eckol	C_18_H_12_O_9_		3.22	371.0409	0	- H
18	ES-	6,6′-Bieckol	C_36_H_22_O_18_		3.39	741.0728	−0.7	- H
19	ES-	Phlorofucofuroeckol A	C_30_H_18_O_14_		4.73	601.063	1.1	- H, +HCOO
20	ES-	Taurocholic acid	C_26_H_45_NO_7_S		8.92	560.2913	2.5	+HCOO
21	ES-	(−)-Abietic acid	C_20_H_30_O_2_		9.22	301.218	2.5	- H
22	ES-	Stigmasta-3α,5α-diol-3-*O*-β-d-glucopyranoside tetracetate		C_43_H_70_O_11_	9.98	807.4919	2.4	+HCOO
23	ES-	Neoabietic acid	C_20_H_30_O_2_		10.53	301.2179	1.9	- H, +HCOO
24	ES-	Pyrophaeophorbide A	C_33_H_34_N_4_O_3_		11.98	533.2551	−1.3	- H
25	ES-	Methyl eicosanoate	C_21_H_42_O_2_		13.2	371.3164	−0.8	+HCOO

### 3.5 Antiviral effects

**Fig. 3:**
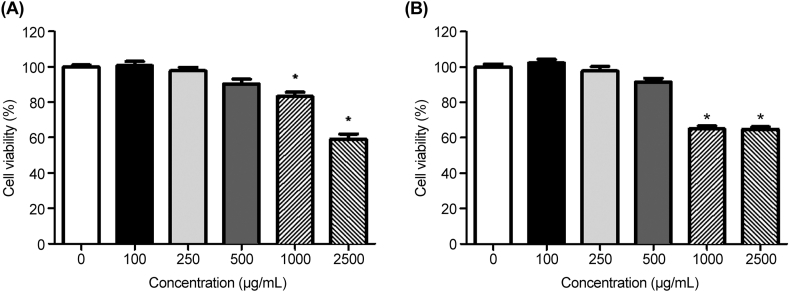
Cytotoxicity of *Ecklonia cava* extract on human liver cancer (HepG2) cell line. HepG2 cells were treated with hot water (A) or 70 % ethanol (B) *E. cava* extracts at the indicated concentrations for 48 h, and cell viability was evaluated using the CCK-8 assay. Data are presented as the mean ± SD of three determinations. *p < 0.05 vs. untreated control.

A cell cytotoxicity assay was conducted using CCK-8, as cytotoxicity could reduce the quantity of HAV RNA, which may be induced at high extract concentrations. HepG2 cells were cultured for 48 h and treated with the two extracts at various concentrations (100, 250, 500, 1000, and 2500 μg/mL) (Fig. 3). No significant changes were observed in the viability of HepG2 cells up to extract concentrations of 500 μg/mL. However, at higher concentrations, these extracts induced greater apoptosis compared with that in the negative control (P < 0.05). Therefore, we conducted antiviral activity experiments at concentrations <500 μg/mL. Kwon et al. [17] reported that phlorofucofuroeckol A and dieckol blocked viral binding to porcine epidemic cells and inhibited replication. Dieckol, a phlorotannin found in *E. cava*, was used as an indicator.

**Fig. 4:**
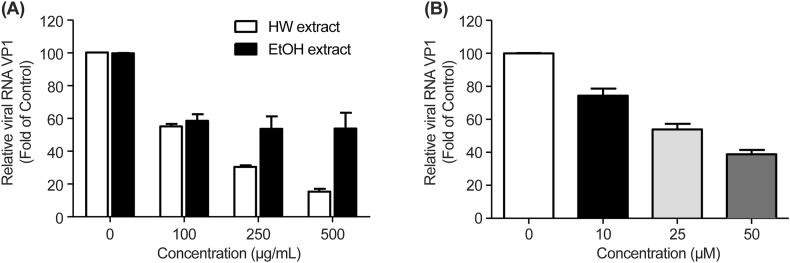
Effects of *Ecklonia cava* extracts and dieckol contribute to restricted hepatitis A virus (HAV) replication. HepG2 cells were infected with HAV and treated with different concentration of hot water or 70 % ethanol extracts (A) and dieckol (B). HAV RNA levels were assessed using qPCR.

HepG2 cells infected with HAV were treated with hot water extract, ethanol extract, and dieckol at various concentrations. Then, HAV mRNA levels were determined relative to those of β-actin using real-time qPCR. Increasing concentrations of *E. cava* extracts decreased the HAV RNA levels in HAV-infected cells (Fig. 4). Additionally, we found that the hot water extract reduced the HAV RNA levels to a greater extent than did the ethanol extract. These results are in contrast with those indicating that the ethanol extract had a higher dieckol extraction efficiency. This suggests that phlorotannins, such as dieckol, as well as other compounds from crude extracts may be responsible for the anti-HAV activities observed. Ogawa et al. [22] and Win et al. [32] reported that zinc in rice-koji miso, which increases GRP78 levels, inhibits HAV replication. On the contrary, Ha et al. [49] reported that many viruses use GRP78 for viral infection and production, and therefore it can be explored as an antiviral target. Madhava et al. [50] reported that phytochemicals from natural products exhibit anti-cancer effects by inhibiting GRP78. GRP78 performs a variety of functional roles, and it is important to understand the relationship between HAV and cells. However, in this study, we have not conducted experiments on the association with GRP78. It would be interesting to investigate the antiviral activity and underlying molecular mechanisms for the individual compounds identified in this study.

## 4 Conclusions

Natural aquatic environments provide various biological activators. In this study, we investigated the antioxidant activity of *E. cava* extracts to identify bioactive phenolic compounds, such as phlorotannins, including phloroeckol, phlorofurofucoeckol A, dieckol, and bieckol derivatives. We also confirmed the antiviral activity of *E. cava* extracts against HAV in HepG2 cells. *E. cava* exhibited high antiviral and antioxidant activities. These findings highlight the potential of this perennial plant as a biomedicine. However, further research is required to isolate and identify antioxidant and antiviral components from these active extracts. Moreover, the mechanisms underlying the inhibition of viral replication by the components warrants further examination.

## Funding

This work was supported in part by grant KE2203-1-1 from the 10.13039/501100003722World Institute of Kimchi, Gwangju, South Korea, and the Priority Research Centres Program through the 10.13039/501100003725National Research Foundation of Korea funded by the 10.13039/501100002701Ministry of Education, Science, and Technology (project no. 2020R1F1A1071038).

## Data availability statement

Data included in article/supplementary material/referenced in the article.

## Ethics approval and consent to participate

Not applicable.

## Consent for publication

Not applicable.

## CRediT authorship contribution statement

**Ye-Sol Kim:** Writing – original draft, Formal analysis. **Ki An Kim:** Writing – original draft, Formal analysis. **Hye-Young Seo:** Writing – original draft, Supervision, Funding acquisition, Formal analysis. **Sung Hyun Kim:** Writing – original draft, Formal analysis. **Hee Min Lee:** Writing – review & editing, Writing – original draft, Project administration, Funding acquisition, Formal analysis, Conceptualization.

## Declaration of competing interest

The authors declare the following financial interests/personal relationships which may be considered as potential competing interests:Hye-Young Seo reports financial support provided by the 10.13039/501100003722World Institute of Kimchi. Hee Min Lee reports financial support provided by the 10.13039/501100003725National Research Foundation of Korea.

## References

[1] Hwang E.K., Gong Y.G., Hwang I.K., Park E.J., Park C.S. (2013). Cultivation of the two perennial brown algae *Ecklonia cava* and *E. Stolonifera* for abalone feeds in Korea. J. Appl. Phycol..

[2] Yoon J.Y., Choi H., Jun H.S. (2017). The effect of phloroglucinol, A component of *Ecklonia cava* extract, on hepatic glucose production. Mar. Drugs.

[3] Kang J.I., Kim S.C., Kim M.K., Boo H.J., Jeon Y.J., Koh Y.S. (2012). Effect of dieckol, a component of *Ecklonia cava*, on the promotion of hair growth. Int. J. Mol. Sci..

[4] Kang N.J., Koo D.H., Kang G.J., Han S.C., Lee B.W., Koh Y.S. (2015). Dieckol, a component of *Ecklonia cava*, suppresses the production of MDC/CCL22 via down-regulating STAT1 pathway in interferon-ɣ stimulated HaCaT human keratinocytes. Biomol Ther (Seoul)..

[5] Park E.Y., Choi H., Yoon J.Y., Lee I.Y., Seo Y., Moon H.S. (2015). Polyphenol-rich fraction of *Ecklonia cava* improves nonalcoholic fatty liver disease in high fat diet-fed mice. Mar. Drugs.

[6] Ale M.T., Mikkelsen J.D., Meyer A.S. (2011). Important determinants for fucoidan bioactivity: a critical review of structure-function relations and extraction methods for fucose-containing sulfated polysaccharides from brown seaweeds. Mar. Drugs.

[7] Wijesekara I., Yoon N.Y., Kim S.K. (2010). Phlorotannins from *Ecklonia cava* (Phaeophyceae): biological activities and potential health benefits. Biofactors.

[8] Ahn G.N., Kim K.N., Cha S.H., Song C., Lee J., Heo M. (2007). Antioxidant activities of phlorotannins purified from *Ecklonia cava* on free radical scavenging using ESR and H2O2-mediated DNA damage. Eur. Food Res. Technol..

[9] Lee S.H., Yong-Li L., Karadeniz F., Kim M.M., Kim S.K. (2009). α-glucosidase and α-amylase inhibitory activities of phloroglucinal derivatives from edible marine brown alga, *Ecklonia cava*. J. Sci. Food Agric..

[10] Zaidi N.A., Hamid A.A.A., Hamid T.H.A.T. (2017). Lactic acid bacteria with antimicrobial properties isolated from the intestines of Japanese quail (*Coturnix japonica*). Galeri warisan Sains.

[11] Montero L., Sánchez-Camargo A.P., García-Cañas V., Tanniou A., Stiger-Pouvreau V., Russo M. (2016). Anti-proliferative activity and chemical characterization by comprehensive two-dimensional liquid chromatography coupled to mass spectrometry of phlorotannins from the brown macroalga *Sargassum muticum* collected on North-Atlantic coasts. J. Chromatogr. A..

[12] Cho S., Yang H., Jeon Y.J., Lee C.J., Jin Y.H., Baek N.I. (2012). Phlorotannins of the edible brown seaweed *Ecklonia cava* Kjellman induce sleep via positive allosteric modulation of gamma-aminobutyric acid type A-benzodiazepine receptor: a novel neurological activity of seaweed polyphenols. Food Chem..

[13] Shin H.C., Hwang H.J., Kang K.J., Lee B.H. (2006). An antioxidative and antiinflammatory agent for potential treatment of osteoarthritis from *Ecklonia cava*. Arch Pharm. Res. (Seoul).

[14] Lee S.H., Jeon Y.J. (2013). Anti-diabetic effects of brown algae derived phlorotannins, marine polyphenols through diverse mechanisms. Fitoter.

[15] Kang M.C., Wijesinghe W.A.J.P., Lee S.H., Kang S.M., Ko S.C., Yang X. (2013). Dieckol isolated from brown seaweed *Ecklonia cava* attenuates type II diabetes in db/db mouse model. Food Chem. Toxicol..

[16] Park J.Y., Kim J.H., Kwon J.M., Kwon H.J., Jeong H.J., Kim Y.M. (2013). Dieckol, a SARS-CoV 3CL(pro) inhibitor, isolated from the edible brown algae *Ecklonia cava*. Bioorg. Med. Chem..

[17] Kwon H.J., Ryu Y.B., Kim Y.M., Song N., Kim C.Y., Rho M.C. (2013). In vitro antiviral activity of phlorotannins isolated from *Ecklonia cava* against porcine epidemic diarrhea coronavirus infection and hemagglutination. Bioorg. Med. Chem..

[18] Yang H.K., Jung M.H., Avunje S., Nikapitiya C., Kang S.Y., Ryu Y.B. (2018). Efficacy of algal *Ecklonia cava* extract against viral hemorrhagic septicemia virus (VHSV). Fish Shellfish Immunol..

[19] Catarino M.D., Silva A.M.S., Mateus N., Cardoso S.M. (2019). Optimization of phlorotannins extraction from *Fucus vesiculosus* and evaluation of their potential to prevent metabolic disorders. Mar. Drugs.

[20] Koivikko R., Loponen J., Pihlaja K., Jormalainen V. (2007). High–performance liquid chromatographic analysis of phlorotannins from the brown alga *Fucus vesiculosus*. Phytochem. Anal..

[21] Tierney M.S., Soler–Vila A., Rai D.K., Croft A.K., Brunton N.P., Smyth T.J. (2014). UPLC–MS profiling of low molecular weight phlorotannin polymers in *Ascophyllum nodosum, Pelvetia canaliculata* and *Fucus spiralis*. Metabolomics.

[22] Ogawa M., Kanda T., Suganami A., Nakamoto S., Win N.N., Tamura Y. (2019). Antiviral activity of zinc sulfate against hepatitis A virus replication. Future Virol..

[23] Hu X., Collier M.G., Xu F. (2020). Hepatitis A outbreaks in developed countries: detection, control, and prevention. Foodborne Pathog. Dis..

[24] Herzog C., van Herck K., van Damme P. (2021). Hepatitis A vaccination and its immunological and epidemiological long-term effects – a review of the evidence. Hum. Vaccin. Immunother..

[25] Güler O., Polat R., Karaköse M., Çakılcıoğlu U., Akbulut S. (2021). An ethnoveterinary study on plants used for the treatment of livestock diseases in the province of Giresun (Turkey). South Afr. J. Bot..

[26] Selvi S., Polat R., Çakılcıoğlu U., Celep F., Dirmenci T., Ertuğ Z.F. (2022). An ethnobotanical review on medicinal plants of the Lamiaceae family in Turkey. Turk. J. Bot..

[27] Battistini R., Rossini I., Ercolini C., Goria M., Callipo M.R., Maurella C. (2019). Antiviral activity of essential oils against hepatitis A virus in soft fruits. J. Food Environ. Virol..

[28] Sánchez G., Aznar R. (2015). Evaluation of natural compounds of plant origin for inactivation of enteric viruses. Food Environ. Virol..

[29] Joshi S.S., Su X., D'Souza D.H. (2015). Antiviral effects of grape seed extract against feline calicivirus, murine norovirus, and hepatitis A virus in model food systems and under gastric conditions. Food Microbiol..

[30] Su X., D'Souza D.H. (2011). Grape seed extract for control of human enteric viruses. Appl. Environ. Microbiol..

[31] Hamdy A.H., Mettwally W.S., El Fotouh M.A., Rodriguez B., El-Dewany A.I., El-Toumy S.A. (2012). Bioactive phenolic compounds from the Egyptian Red Sea seagrass *Thalassodendron ciliatum*. Z. Naturforsch., C: J. Biosci..

[32] Win N.N., Kanda T., Nakamoto S., Moriyama M., Jiang X., Suganami A. (2018). Inhibitory effect of Japanese rice-koji miso extracts on hepatitis A virus replication in association with the elevation of glucose-regulated protein 78 expression. Int. J. Med. Sci..

[33] Folin O., Denis W. (1912). On phosphotungstic-phosphomolybdic compounds as color reagents. J. Biol. Chem..

[34] Davis W.B. (1947). Determination of flavanones in citrus fruits. Anal. Chem..

[35] Blois M.S. (1958). Antioxidant determinations by the use of a stable free radical. Nature.

[36] Re R., Pellegrini N., Proteggente A., Pannala A., Yang M., Rice-Evans C. (1999). Antioxidant activity applying an improved ABTS radical cation decolorization assay. Free Radic. Biol. Med..

[37] Lee S.H., Kang M.C., Moon S.H., Jeon B.T., Jeon Y.J. (2013). Potential use of ultrasound in antioxidant extraction from *Ecklonia cava*. ALGAE.

[38] Liu X., Luo G., Wang L., Yuan W. (2019). Optimization of antioxidant extraction from edible brown algae Ascophyllum nodosum using response surface methodology. Food Bioprod. Process..

[39] Ertani A., Francioso O., Tinti A., Schiavon M., Pizzeghello D., Nardi S. (2018). Evaluation of seaweed extracts from *Laminaria* and *Ascophyllum nodosum* spp. as biostimulants in *Zea mays* L. using a combination of chemical, biochemical and morphological approaches. Front. Plant Sci..

[40] Hesse M., Meier H., Zeeh B. (2005). Spektroskopische Methoden in der Organischen Chemie.

[41] Gisbert M., Sineiro J., Moreira R. (2022). Influence of oxidation and dialysis of phlorotannins on bioactivity and composition of ultrasound-assisted extracts from *Ascophyllum nodosum*. Mar. Drugs.

[42] Ford L., Theodoridou K., Sheldrake G.N., Walsh P.J. (2019). A critical review of analytical methods used for the chemical characterisation and quantification of phlorotannin compounds in brown seaweeds. Phytochem. Anal..

[43] González-Colunga D., Antunes-Ricardo M., Gutiérrez-Uribe J.A. (2019). Bioactivity-guided identification of anti-AHPND (acute hepatopancreatic necrosis disease) metabolites of *Ecklonia arborea*. J. Appl. Phycol..

[44] Wu Y., Gao H., Wang Y., Peng Z., Guo Z., Ma Y., Zhang R., Zhang M., Wu Q., Xiao J., Zhong Q. (2022). Effects of different extraction methods on contents, profiles, and antioxidant abilities of free and bound phenolics of *Sargassum polycystum* from the South China Sea. J. Food Sci..

[45] Bhuvana P., Sangeetha P., Anuradha V., Ali M.S. (2019). Spectral characterization of bioactive compounds from microalgae: *N. Oculata* and *C. vulgaris*. Biocatal. Agric. Biotechnol..

[46] Dai H., Xu X., Li W., Fu X., Han W., Li G. (2023). Investigating the vital role of the identified abietic acid from *Helianthus annuus* L. calathide extract against hyperuricemia via human embryonic kidney 293T cell model. Molecules.

[47] Park S., Kim J.Y., Kwon H.C., Jang D.S., Song Y.J. (2022). Antiviral activities of ethyl pheophorbides a and b isolated from *Aster pseudoglehnii* against influenza viruses. Molecules.

[48] Xun Z., Lin J., Yu Q., Liu C., Huang J., Shang H. (2021). Taurocholic acid inhibits the response to interferon-α therapy in patients with HBeAg-positive chronic hepatitis B by impairing CD8^+^ T and NK cell function. Cell. Mol. Immunol..

[49] Ha D.P., van Krieken R., Carlos A.J., Lee A.S. (2020). The stress-inducible molecular chaperone GRP78 as potential therapeutic target for coronavirus infection. J. Infect..

[50] Madhavan S., Nagarajan S. (2020). GRP78 and next generation cancer hallmarks: an underexplored molecular target in cancer chemoprevention research. Biochimie.

